# A Population-Based Study of Attention Deficit/Hyperactivity Disorder Symptoms and Associated Impairment in Middle-Aged Adults

**DOI:** 10.1371/journal.pone.0031500

**Published:** 2012-02-08

**Authors:** Debjani Das, Nicolas Cherbuin, Peter Butterworth, Kaarin J. Anstey, Simon Easteal

**Affiliations:** 1 John Curtin School of Medical Research, The Australian National University, Canberra, Australian Capital Territory, Australia; 2 Centre for Research on Ageing, Health and Wellbeing, Australian National University, Canberra, Australian Capital Territory, Australia; Alexander Flemming Biomedical Sciences Research Center, Greece

## Abstract

Attention deficit/hyperactivity disorder (ADHD) is the most prevalent childhood psychiatric condition. It frequently persists into adulthood and can have serious health and other adverse consequences. The majority of previous adult ADHD studies have focused on young adults so that relatively little is known about ADHD symptoms and their effects in mid and late life. In addition, effects of subclinical levels of attention deficit and hyperactivity have not been studied in detail. In this study we investigated ADHD symptoms and related impairment in a large population-based sample of middle-aged Australian adults (n = 2091; 47% male). Applying the WHO adult ADHD Self Report Screener (ASRS) we observed that 6.2% of participants had scores that were previously associated with ADHD diagnosis. No significant gender difference in the distribution of ASRS scores was observed. Multiple regression analyses indicated strong positive correlations between symptoms of ADHD and depression/anxiety and significant negative associations (*p*<0.01) with employment, financial stress, relationship quality, health and well-being measures in this age group. Importantly, associations were highly significant even when few ADHD symptoms were reported. Compared to the hyperactivity component, the inattention trait was particularly strongly associated and remained significant after controlling for depression/anxiety symptoms. Our study confirms previous findings and significantly adds to existing literature especially for an age-group that has not been well-studied. Our results suggest that ADHD symptoms continue to be associated with ill-health and functional impairment in mid-life and are, therefore, likely to be a major, previously unrecognized source of late-life morbidity with associated social and economic costs. Thus, there is a compelling need for better understanding and development of age-appropriate approaches to the diagnosis and treatment of ADHD in mid- to late-life.

## Introduction

Attention Deficit/Hyperactivity Disorder (ADHD) is a treatable neuropsychiatric disorder characterized by inattention, excessive motor activity and impulsivity [1], which begins in childhood and can persist across the lifespan. However, symptom patterns change with age and become less obvious and specific in adults [2]. Most adults predominantly exhibit problems with inattention, which manifest as disorganization, forgetfulness, unreliability, and difficulty in planning, task completion, task shifting and time management [2], [3]. These symptoms adversely affect multiple life domains with serious negative impact on functioning in day-to-day life. Adult ADHD is also associated with a wide range of other disorders, such as mood and substance use disorders [2], [4] and its detrimental effect result in substantial public health and other costs [5]–[9]. A recent meta-analysis study reported that the prevalence estimate of adult ADHD is 2.5% with a significant interaction effect of age and gender on the estimate [10]. Earlier studies had reported estimates between 1.0 and 7.3% [11]–[22]. Although the prevalence of ADHD is thought to decrease with age; these estimates suggest that ADHD is one of the most common adult psychiatric disorders. Moreover, the true population prevalence of the disorder is likely to be higher since under-reporting of symptom severity is common and because of problems with current diagnostic methods applied to adults [2], [23], [24].

Clinical diagnosis of ADHD typically follows categorical definitions codified in The Diagnostic and Statistical Manual of Mental Disorders, 4^th^ edition, Text Revision (DSM-IV-TR) [25]. However, accumulating evidence strongly supports ADHD symptoms as being dimensional rather than categorical with the disorder existing at the extremes of symptom distributions [26]. It has been suggested that the dimensional approach is likely to capture clinical and research data far more effectively than the categorical approaches currently used [27]. ADHD symptoms are also quite common; Arcos-Burgos et al. (2007) [28] reported that nearly 60% of the general population displays some symptoms of inattention and hyperactivity. Furthermore, individuals with ADHD symptoms who never meet the DSM threshold for diagnosis are also significantly impaired and have a milder form of the disorder [29], [30]. However, effects of few and/or mild symptoms have not been studied in detail.

While awareness of adult ADHD has increased over the last decade, we know relatively little about the disorder in older adults. Few studies [10], [21], [22], [31] have included individuals over the age of 45 years when estimating prevalence rate in adult populations. Guldberg-Kjar and Johansson (2009) [32] studied self-rated ADHD symptoms in 65–80 year old Swedish adults and reported a prevalence rate of 3.3%, which is comparable to that in the younger age-groups. It has also been suggested that older adults with ADHD suffer from cumulative consequence of the disorder over the lifespan, which affects quality of life at late-age [33]. Thus, persistence of ADHD and its adverse consequences into late life has important public health implications. Since ADHD is a treatable condition, greater emphasis on its diagnosis and treatment in older people might result in sizeable reductions in late-age morbidity.

There has been increasing recognition that midlife represents a critical period where interventions could lead to healthier and more productive ageing. However, this age-group has been largely neglected with respect to mental health research as much of the focus is on the young or the elderly. In this study we investigated the impact of self-reported ADHD symptoms on a range of measures of health, work, social life, relationship, well-being and anxiety/depression symptoms in a large population-based sample of middle-aged adults. We used the World Health Organization's adult ADHD Self Report Screener (ASRS) [34] questionnaire (widely used in epidemiological setting) and followed methods reported by Kessler et al. (2005; 2007) [34], [35] to score individuals, which allowed us to identify individuals with few and/or mild symptoms. Furthermore, we investigated the relative impact of the symptom dimensions of inattention and hyperactivity on functional impairment. These methods allowed us to provide a comprehensive view of the functional impairments experienced by middle-aged adults with inattention and hyperactivity symptoms that were not accounted for by co-occurring depression/anxiety symptoms.

## Methods

### Ethics statement

All participants gave written informed consent to be included in the Personality and Total health (PATH) project. The study was approved by the Human Research Ethics Committee of The Australian National University.

### Participants

The study sample was drawn from the middle-aged cohort of the PATH Through Life Project, which is a longitudinal study of mental health and ageing [36]. Participants were residents of the city of Canberra and the adjacent town of Queanbeyan in south-eastern Australia and were recruited randomly from the electoral roll (which provides a good representative population sample because enrolment to vote is a legal requirement for all adult Australian citizens). Further information on this cohort can be found in the report by Anstey et al. (2011) [36]. Respondents are interviewed every four years, with three waves of data collection completed to date. Participants were surveyed for potential risk factors and moderators of mental health.

The present study used data from the third wave of assessment (the ASRS scale was introduced in this wave), which included 2179 individuals aged 47–54 years (mean age 50.7±1.5 years). Twenty-five participants (1.2%) failed to complete the ASRS questionnaire. These non-responders did not significantly (p>0.05) differ from the responders with respect to any of the sociodemographic variables. A further 63 participants who reported having epilepsy, brain tumor/infection or severe head injury were excluded, leaving a sample of n = 2092 (47% male) available for this investigation. One participant reported being prescribed Dexamphetamine, which is used for treating ADHD. Inclusion or exclusion of this participant did not affect the result of our analyses (only results with this participant excluded are reported).

### Measures

All self-report measures used in this study were obtained using validated instruments. The ASRS was developed by the World Health Organization and the short form of the screener consists of a checklist of six questions regarding symptoms of ADHD based on the diagnostic criteria of DSM-IV-TR [34], [35]. Each item requires respondents to rate on a five-point response scale from “never” [0] to “very often” [4] how frequently a particular symptom of ADHD occurred over the past six months. A summary score with a theoretical range of 0–24 was obtained as an equally weighted sum of response scores for all questions. Higher scores indicate increased risk of ADHD. Based on the classification methods recommended by Kessler et al (2005, 2007) [34], [35] participants were grouped into: (i) binary categories using a cutoff score of 13 (scores 14–24 being indicative of possible ADHD) or (ii) four strata with the following score ranges: 0–9 (stratum I), 10–13 (stratum II), 14–17 (stratum III) and 18–24 (stratum IV). To explore these dimensions separately we used an equally weighted sum of response scores for the first four items of the screener related to inattention as the Inattention Trait Score (ITS) and of response scores for the last two items related to hyperactivity as the Hyperactivity Trait Score (HTS).

Categorical measures related to employment status, financial stress and married/cohabiting relationship were generated from responses to questions such as “How would you describe your current employment status?”, “Have you or your family had to go without things you really needed in the last year because you were short of money?”, “Are you currently in a relationship with someone?” and “How many times have you been married or lived in a de facto relationship?”. The 7-item short form of Dyadic Adjustment Scale (DAS-7) was used to measure adjustment in a married or cohabiting relationship. An equally weighted sum of the response scores for the seven items was used the DAS-7 total score [37]. The Lubben social network scale (LSNS) has been used extensively to assess social integration and to screen for social isolation. We used the abbreviated version of the scale with six items (LSNS-6; [38]), three of which relate to kinship ties and the remaining three to non-kin ties. The total scale score was an equally weighted sum of response scores for all the items (range 0–30).

Health-related quality of life was measured using RAND-12, which contains 12 items from the eight scales of the RAND-36 Health Status Inventory [39]. A set of six questions creates the physical health composite while the remaining six creates the mental health composite. Each scale is scored using a one parameter Rasch model based on Item Response Theory [39]. Depression and anxiety symptoms were assessed using items from the section covering mood and panic disorders in the Patient Health Questionnaire (PHQ). This is a short version of the patient questionnaire component of the Primary Care Evaluation of Mental Disorders (PRIME-MD) instrument [40]. We generated measures of depression/anxiety- related disorders from the nine items related to depression symptoms [rated on a 4-point scale from “not at all” (1) to “nearly every day” (4)] and the seven items related to anxiety symptoms [rated on a 3-point scale from “not at all” (1) to “more than half the days” (3)] following the coding algorithm provided in the PHQ instruction manual. Participants also reported on the use of medication for anxiety and depression.

The three separate components of subjective well-being – positive affect (reflecting enthusiastic, alert and active feelings), negative affect (reflecting subjective distress and unpleasurable engagement) and life satisfaction [41] were measured using two scales - the Positive and Negative Affect Schedule (PANAS;) [42] and the satisfaction with life scale (SWLS;) [43]. PANAS has 20 items, 10 for each affect type. Responses to each item were scored on a 5-point scale from “very slightly” (1) to “extremely” (5). An equally weighted sum of the response scores for items in each subscale was used as the total scale score (range 10–50). The SWLS has 5 items each scored on a 7-point scale ranging from “strongly agree” (1) to “strongly disagree” (7). The total scale score was an equally weighted sum of the response scores for the five items, with a range of 5–35.

### Statistical analysis

All statistical analyses were conducted using SPSS 18 (Chicago: SPSS Inc.). Means and standard deviations were computed for the ASRS total score, ITS and HTS. Comparisons between males and females were performed using Student's t-tests and Pearson's Chi-square tests for the continuous and categorical ASRS measures respectively. Associations between the symptoms of ADHD and depression/anxiety symptoms, employment status, financial problems, RAND-12, PAS, NAS, SWLS, DAS-7 and LSNS-6 scores were analyzed using linear or logistic regressions as appropriate. Age, sex and total years of education were included as covariates in all models. Depression and anxiety symptoms were included as additional covariates in models as indicated. Regression models were generated by entering the covariates first, followed by the predictors. Change in *R^2^* value between each step and the *p* value associated with the *R^2^* change were noted. For simplicity in interpretation we report results that were significant at the stringent α level of 0.01 instead of the more commonly used level of α = 0.05. However, the majority of results remained significant after applying Bonferroni corrections at an α = 0.05.

## Results

### ADHD symptoms at midlife

**Figure 1:**
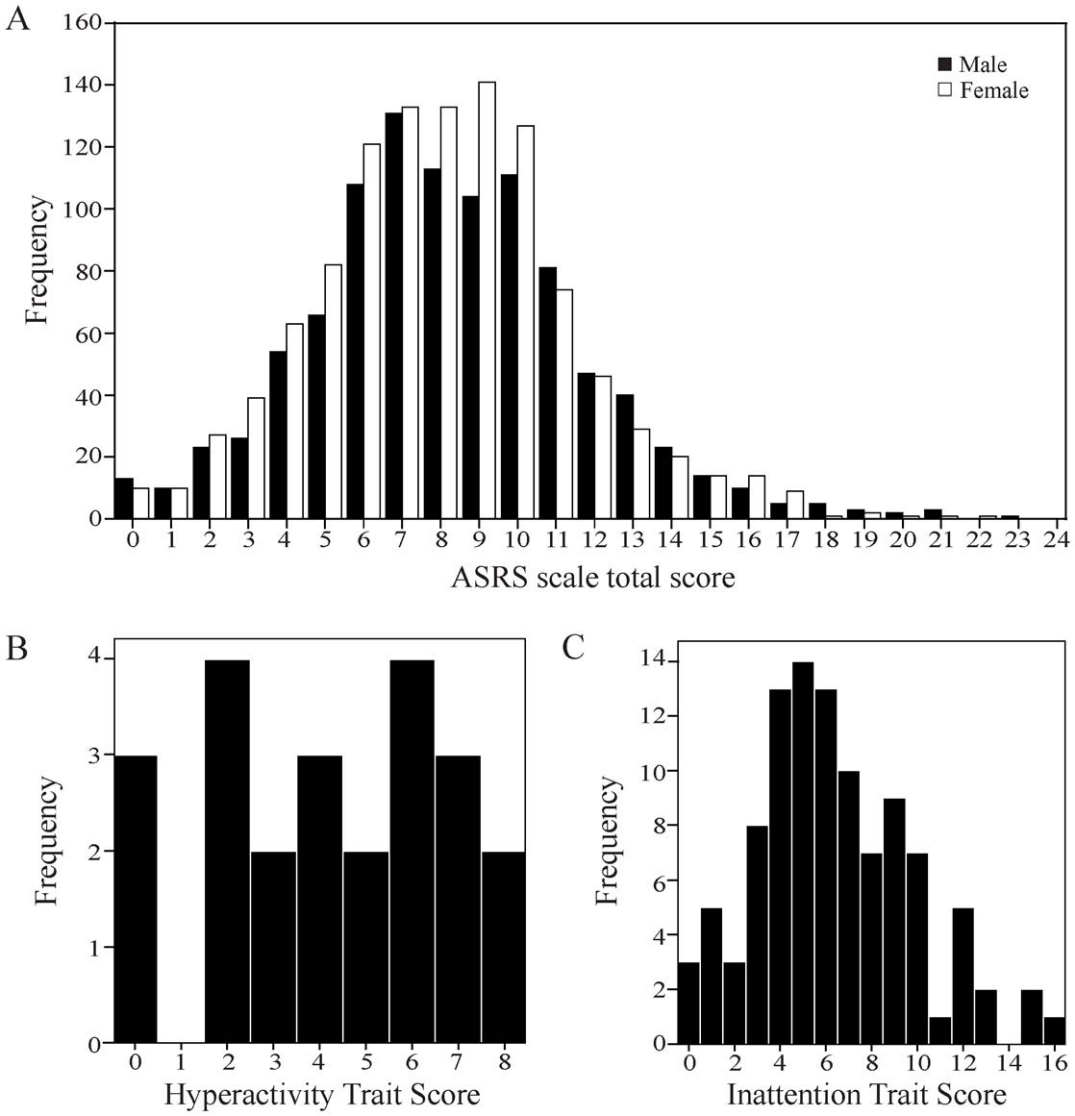
Distribution of ASRS score, HTS and ITS. A) ASRS total score shown separately for males and females. B) HTS shown if responses to all inatention-related ASRS items were “often” or “very often”; C) ITS shown if responses to all hyperactivity-related ASRS items were “often” or “very often”.

For each individual item on the ASRS questionnaire, the frequency of participants responding to each point of the response scale is shown in Table 1. The proportion of participants reporting “often” or “very often” varied for individual items, ranging from 6–17%. The ASRS total score followed an approximate normal distribution in this sample (Figure 1A) with a mean score of 8.2 and a standard deviation of 3.4. To distinguish possible ADHD cases from non-cases at the individual level we applied both the dichotomous and the four-strata classification schemes as suggested by Kessler et al (2007) [35]. Using the dichotomous classification scheme, 6.2% of participants had scores equal to or above the cut-off score of 14, which has been previously associated with ADHD diagnosis. When the four-strata classification scheme was applied, 67.3%, 26.5%, 5.2% and 1% of participants were present in strata I, II, III and IV, respectively. The four-strata classification was reported to have strong concordance with clinical diagnosis with stratum IV most likely and stratum I least likely to contain ADHD cases. Strata II and III represents those with few and/or mild ADHD symptoms and might represent subthreshold cases. We also found no significant difference between males and females in either the continuous or categorical ASRS measures (Table 2).

**Table 1:** Frequency of responses to ASRS items.

ASRS items		Never	Rarely	Sometimes	Often	Very often
*Inattention*						
1.	Trouble wrapping up project	333 (15.9%)	855 (40.9%)	701 (33.5%)	165 (7.9%)	37 (1.8%)
2.	Difficulty getting things in order	390 (18.7%)	1039 (49.7%)	539 (25.8%)	98 (4.7%)	25 (1.2%)
3.	Problems remembering appointments	420 (20.1%)	1075 (51.4%)	479 (22.9%)	95 (4.5%)	22 (1.1%)
4.	Avoid or delay getting started	187 (8.9%)	778 (37.2%)	890 (42.6%)	186 (8.9%)	50 (2.4%)
*Hyperactivity*						
5.	Fidget or squirm hands or feet	369 (17.6%)	741 (35.4%)	629 (30.1%)	263 (12.6%)	89 (4.3%)
6.	Overly active or compelled to do things	463 (22.1%)	802 (38.4%)	575 (27.5%)	181 (8.7%)	70 (3.3%)

ASRS: Adult ADHD Self Report Screener.

**Table 2:** Gender differences in ASRS scores (Mean ± s.d. for continuous variables and frequency for categorical variables shown).

	Males	Females	t/χ^2^	df	*p*
	(n = 993)	(n = 1098)			
ASRS total score	8.3±3.5	8.0±3.3	1.568	2089	0.117
Inattention trait score	5.5±2.7	5.2±2.6	2.250	2089	0.025
Hyperactivity trait score	2.8±1.7	2.8±1.7	−0.359	2089	0.719
ASRS strata			6.947	3	0.074
I (n = 1408)	65.3%	69.1%			
II (n = 555)	28.1%	25.1%			
III (n = 109)	5.2%	5.2%			
IV (n = 20)	1.4%	0.5%			
ASRS category			0.744	1	0.388
0–13 (n = 1963)	93.4%	94.3%			
14–24 (n = 129)	6.6%	5.7%			

ASRS: Adult ADHD Self Report Screener.

To assess ADHD symptom dimensions of inattention and hyperactivity, we calculated inattention and hyperactivity item scores separately. These scores referred to as trait scores (ITS and HTS) were normally distributed in our sample with means and standard deviations of 5.3±2.7 and 2.8±1.7, respectively. There was no significant difference between males and females for either trait, although a non-significant trend towards higher ITS in males was observed (Table 2). While ITS was significantly correlated to HTS (Pearson's *r* = 0.199, *p*<0.001), we observed that even when participants reported “often” or “very often” for all inattention items (n = 23) their HTS scores were distributed over the entire range of the scale (Figure 1B). A similar distribution was observed for hyperactivity items (n = 103) and ITS scores (Figure 1C). Hence, for all subsequent analyses we present results separately for ITS and HTS as continuous measures of ADHD symptoms. We also present results for the four ASRS strata as a categorical classification of ADHD symptoms, as recommended by Kessler et al (2007) [35], to enable our results to be compared with clinical studies.

### ADHD symptoms are highly correlated with symptoms of depression and anxiety

We observed significant associations between ADHD symptoms and both depression and anxiety symptoms (Table 3). Compared to the stratum I of the ASRS, a significantly higher proportion of participants in all the other strata report the presence of depression or anxiety symptoms. Use of anxiety and depression medication was also higher in strata II, III and IV compared to stratum I. Further, after adjusting for age, sex and years of education, the odds of reporting depression or anxiety symptoms increased significantly with increase in either ITS and HTS (Table 4). The odds are also significantly higher for the ASRS strata II, III and IV compared to stratum I (Table 5). These results demonstrate that there is substantial overlap between ADHD and depression/anxiety symptoms in this age group. This is also true when few and/or mild ADHD symptoms were reported. We did not find any gender difference in the association between ADHD and depression/anxiety symptoms.

**Table 3:** Associations between ASRS strata and categorical classification of depression and anxiety symptoms.

	ASRS strata				χ^2^	df	*p*
	I	II	III	IV			
	(n = 1407)	(n = 551)	(n = 109)	(n = 20)			
BPHQ depression categories					217.922	9	**<0.001**
No depression (n = 1800)	90.8%	82.2%	54.1%	55.0%			
Subsyndromal (n = 149)	5.8%	8.0%	18.3%	20.0%			
Minor (n = 71)	2.5%	5.1%	7.3%	0.0%			
Major (n = 66)	0.9%	4.7%	20.2%	25.0%			
BPHQ anxiety symptoms					132.645	3	**<0.001**
Absent (n = 1995)	97.8%	93.9%	76.1%	75.0%			
Present (n = 96)	2.2%	6.1%	23.9%	25.0%			
Anxiety/Depression medication use					70.725	3	**<0.001**
No (n = 1832)	91.8%	81.9%	73.4%	65.0%			
Yes (n = 251)	8.2%	18.1%	26.6%	35.0%			

p<0.01 shown in bold.

BPHQ: Brief Patient Health Questionnaire.

**Table 4:** Logistic regression model with ITS and HTS as predictors.

	Inattention trait score			Hyperactivity trait score			
	B	*p*	OR (95% CI)	B	*p*	OR (95% CI)	R^2^
Depression^a^	0.239	**<0.001**	1.27 (1.21, 1.33)	0.117	**0.003**	1.12 (1.04, 1.21)	0.114
Anxiety^a^	0.265	**<0.001**	1.30 (1.21, 1.40)	0.203	**0.001**	1.23 (1.09, 1.38)	0.116
Employed^b^	−0.076	0.014	0.93 (0.87, 0.99)	−0.049	0.316	0.95 (0.87, 1.05)	0.058
Financial stress^c^	0.101	**<0.001**	1.11 (1.06, 1.16)	0.098	**0.005**	1.10 (1.03, 1.18)	0.042
Partner present	−0.038	0.086	0.96 (0.92, 1.10)	0.067	0.058	1.07 (1.00, 1.15)	0.016
Separated^d^	0.029	0.099	1.03 (1.00, 1.07)	0.049	0.068	1.05 (1.00, 1.11)	0.010

Models adjusted for age, sex and total years of education.

p<0.01 shown in bold.

a “symptoms absent” is the reference category.

b “not currently employed” is the reference category.

c “financial stress absent” is the reference category.

d “never been separated or divorced” is the reference category.

OR: Odds Ratio; CI: Confidence Interval; R^2^ = Nagelkerke R^2^.

**Table 5:** Logistic regression models with ASRS strata as predictor.

	ASRS strata^a^									
	II			III			IV			
	B	*p*	OR (95% CI)	B	*p*	OR (95% CI)	B	*p*	OR (95% CI)	R^2^
Depression^b^	0.814	**<0.001**	2.26 (1.70, 3.00)	2.180	**<0.001**	8.85 (5.80, 13.50)	2.280	**<0.001**	9.78 (3.86, 24.81)	0.108
Anxiety^b^	1.072	**<0.001**	2.92 (1.76, 4.81)	2.645	**<0.001**	14.08 (7.98, 24.84)	2.743	**<0.001**	15.53 (5.24, 49.00)	0.128
Employed^c^	0.045	0.824	1.05 (0.71, 1.55)	−1.167	**<0.001**	0.31 (0.18, 0.53)	−1.354	0.024	0.26 (0.08, 0.84)	0.071
Financial stress^d^	0.538	**<0.001** f	1.71 (1.32, 2.21)	0.974	**<0.001**	2.65 (1.70, 4.12)	0.906	0.089	2.47 (0.87, 7.03)	0.038
Partner present	−0.123	0.340	0.89 (0.69, 1.14)	−0.069	0.790	0.93 (0.56, 1.55)	0.072	0.911	1.07 (0.31, 3.74)	0.012
Separated^e^	0.129	0.210	1.14 (0.93, 1.39)	0.533	**0.008**	1.70 (1.15, 2.52)	1.048	0.029	2.84 (1.11, 7.32)	0.013

Models adjusted for age, sex and total years of education.

p<0.01 shown in bold.

a ASRS strata I is the reference category.

b “symptoms absent” is the reference category.

c “not currently employed” is the reference category.

d “financial stress absent” is the reference category.

e “never been separated or divorced” is the reference category.

f trend observed after adjusting for depression and anxiety symptoms.

OR: Odds Ratio; CI: Confidence Interval; R^2^ = Nagelkerke R^2^.

### ADHD symptoms correlate with employment status and financial stress

In our sample 8.0% and 16.7% of participants reported being currently unemployed and having financial problems respectively. ITS and HTS do not significantly alter the odds of being currently employed although there was a trend towards lower odds of being employed for higher ITS (Table 4). In addition, individuals who reported having financial problems had significantly higher scores for both ITS and HTS (Table 4). Participants in strata II and III had significantly higher odds of experiencing financial problems while participants in ASRS stratum III also had lower odds of being employed compared to stratum I (Table 5). A similar pattern was detected for stratum IV, which did not reach significance, possibly reflecting the relatively small number of participants in this stratum.

### Inattention symptoms significantly affect multiple life domains in mid-life

In our sample 18.1% reported not having a partner and 40.0% reported being separated or being divorced. Neither trait score was significantly associated with having a partner or being separated/divorced (Table 4). Interestingly, individuals who reported two or more marriages/relationships had significantly higher HTS but not ITS when compared to those reporting only one marriage/relationship (*p*<0.01). No significant difference was observed when “never being married or in a relationship” was included in the analysis. Significant associations are also observed between ITS and measures of cohabiting/marital relationship adjustment (DAS-7), health related quality of life (RAND-12), subjective well-being (PANAS and SWLS), social interactions (LSNS-6) and affect (PAS and NAS; Table 6). However, only the health and affect measures are significantly associated with HTS. With the exception of NAS, all the other variables are negatively associated with ADHD symptoms. When ASRS strata was the predictor variable, the results are very similar to those obtained for the ITS (Tables 5 and 6). Interestingly, associations were highly significant for both strata II and III. However, in some cases the difference between strata I and IV did not reach statistical significance, probably because there are very few individuals in stratum IV.

**Table 6:** Multiple regression models with inattention and hyperactivity trait scores or ASRS strata as predictors.

	ADHD symptoms					ASRS strata^a^						
	ITS		HTS			II		III		IV		
	β	*p*	β	*p*	R^2^ (change)	β	*p*	β	*p*	β	*p*	R^2^ (change)
DAS-7	−0.208	**<0.001** c	0.003	0.910	0.045 (0.043)^b^	−0.112	**<0.001** d	−0.172	**<0.001** c	−0.016	0.510	0.039 (0.036)^b^
LSNS-6	−0.144	**<0.001** c	0.016	0.455	0.039 (0.020)^b^	−0.078	**<0.001**	−0.128	**<0.001** c	−0.045	0.037	0.040 (0.021)^b^
RAND-12 Physical	−0.217	**<0.001** c	0.059	**0.006**	0.076 (0.055)^b^	−0.128	**<0.001**	−0.182	**<0.001** d	−0.081	**<0.001**	0.069 (0.047)^b^
RAND-12 Mental	−0.362	**<0.001** c	−0.091	**<0.001**	0.157 (0.150)^b^	−0.239	**<0.001** c	−0.306	**<0.001** c	−0.126	**<0.001**	0.147 (0.140)^b^
PAS	−0.324	**<0.001** c	0.064	**0.002** c	0.122 (0.099)^b^	−0.144	**<0.001**	−0.177	**<0.001**	−0.051	0.017	0.069 (0.046)^b^
NAS	0.298	**<0.001** c	0.160	**<0.001** c	0.133 (0.132)^b^	0.225	**<0.001** c	0.313	**<0.001** c	0.176	**<0.001** c	0.154 (0.152)^b^
SWLS	−0.299	**<0.001** c	−0.005	0.821^d^	0.095 (0.089)^b^	−0.160	**<0.001** c	−0.207	**<0.001** c	−0.097	**<0.001**	0.072 (0.066)^b^

Models adjusted for age, sex and total years of education.

p<0.01 shown in bold.

a ASRS strata I is the reference category.

b significant R^2^ change from previous model.

c significant after adjusting for depression and anxiety symptoms.

d trend observed after adjusting for depression and anxiety symptoms.

ITS: Inattention Trait Score; HTS: Hyperactivity Trait Score; DAS: Dyadic Adjustment Scale; LSNS: Lubben Social Network Scale; PAS: Positive Affect Schedule; NAS: Negative Affect Schedule; SWLS: Satisfaction With Life Scale.

Depression and anxiety symptoms also affect functioning in the domains we investigated and since in our sample ADHD and depression/anxiety symptoms were highly correlated, we examined whether the significant associations reported above were confounded by the presence of depression/anxiety symptoms. We generated regression models controlling for depression and anxiety measures in addition to age, sex and years of education for all variables for which we had previously detected significant associations with ADHD symptoms. Using ITS and HTS as predictors we observed that after controlling for depression and anxiety symptoms, all associations with ITS remain significant, but only scores for positive and negative affect remain significantly associated with HTS (Table 6). When the predictor was ASRS strata, association with all but RAND-12 physical health and PAS scores remain statistically significant (Table 6). The differences were most significant for comparisons between stratum I and stratum III (also stratum II for some variables) but not stratum IV, probably because very few individuals were classified as ASRS stratum IV. These results demonstrate that ADHD symptoms affect health, subjective well-being, ability to interact socially and maintain healthy relationships in middle-aged individuals independent of their depression/anxiety symptoms. This is true even when few ADHD symptoms were reported and impairments were primarily associated with inattention symptoms.

## Discussion

In this study we investigated self-reported inattention and hyperactivity symptoms in a large community-based sample of middle-aged adults. The narrow age range of our sample allowed us to perform focused investigations in this age group without the confounding effects of age. We evaluated multiple symptomatic and functional domains, which allowed us to present a comprehensive picture of impairments associated with ADHD symptoms. We confirm earlier findings from samples of young ADHD adults and additionally report that middle-age individuals experiencing few symptoms are significantly impaired in several domains including work and social life. Our study is thus a significant addition to existing literature especially in an age group that has not been well studied but which is critically important in the context of promoting healthy ageing. While the clinical status of participants could not be confirmed our results clearly demonstrate the substantial burden associated with ADHD symptoms in middle age. Inattention symptoms are associated with significantly more functional impairment than hyperactivity symptoms. While most of our results closely follow the observations made previously in clinical samples of younger age groups, some differences are apparent. Several factors could have contributed to these differences. An obvious factor is that our sample represents an age group not extensively studied for ADHD and associated impairment and hence, there is limited scope for direct comparison with existing studies. In addition, our sample is more representative of the general middle-aged population compared to the referred or convenience samples that have been more widely studied. It is important to note in this context that our results refer specifically to inattention and hyperactivity symptoms and not clinically diagnosed ADHD cases.

The present study did not include diagnostic assessment of participants and was not designed to estimate prevalence rates of ADHD in middle age. Nevertheless, following the dichotomous classification of the ASRS scores 6.2% of our sample fell within the score range that had previously been associated with the diagnosis of ADHD. The ASRS score had high concordance with the clinical status of subjects in validation studies [35]. Our estimate thus provides an indication of the prevalence of ADHD in this population. Furthermore, the proportion of possible ADHD cases in our sample falls within the range of prevalence rates previously reported in adult samples [11]–[22].

### Gender differences

In our sample ADHD symptoms were not significantly different between males and females. This result is contrary to some previous reports, which found significantly higher prevalence of ADHD in males [10], but is consistent with findings from another recent study of a large representative population sample [31]. This difference could be due to the fact that we report ADHD symptoms and not clinically diagnosed cases. However, gender difference in ADHD prevalence is known to be more extreme in pediatric samples compared to adolescent and adult samples. It has been reported that since childhood referrals are initiated by parents and teachers, girls without the hyperactivity component of ADHD are less likely to be clinically diagnosed [44]. In contrast, women experience more internalizing problems than men, which leads to higher rates of self-referrals in adulthood thereby generating more balanced gender distributions in adult samples [45].

We also found no significant gender difference in the association between ADHD symptoms and depression/anxiety scores or measures of functional impairment. This result is consistent with previous findings in clinically referred subjects [46] and suggests that men and women experience ADHD in a similar way.

### Depression and Anxiety

In our sample symptoms of ADHD very often co-occurred with depression and anxiety symptoms. This has been observed in both clinical and population samples of younger adults [20]. Even among adults with few ADHD symptoms (Stratum II), and hence unlikely to meet the threshold for a clinical diagnosis, the associations with both depression and anxiety symptoms were substantial. Also, both ITS and HTS were strongly associated with depression and anxiety symptoms although the association with inattention symptoms were much stronger. Because of the cross-sectional nature of the study we could not ascertain whether depression/anxiety symptoms were secondary to ADHD symptoms. The use of depression or anxiety medication is also significantly higher among individuals with ADHD symptoms, which is likely to contribute to higher medical costs and financial stress.

### General functioning

We observe trends suggesting that ADHD symptoms are negatively associated with full-time employment and positively associated with financial problems. In addition ADHD symptoms are negatively associated with relationship quality, social life, health and well-being. These findings are broadly consistent with studies of adult ADHD patients or follow-up studies on children with ADHD [21], [47]–[49]. We also observe that these associations remain significant even after removing the variance explained by depression and anxiety symptoms. The effect is particularly strong for inattention symptoms. Thus, in the general population, individuals experiencing even few ADHD symptoms have impaired functioning in personal and social domains, which correlates with poor health and well-being. Persistence of these impairments is likely to increase late-life morbidity, a significant public health issue for countries with aging populations.

The main strength of this study was that it was conducted in a large representative epidemiological sample, thereby avoiding the potential biases associated with clinical samples and hence the results are likely to be more generalisable to the middle-aged population. Further, participants in our study represent an age group that has not been well-studied for problems of attention regulation (which have been more widely explored in children or in older adults in relation to dementia). We included assessment of multiple symptomatic and functional domains affected by ADHD to provide a comprehensive description of functional impairment, while earlier studies have largely focused on health status and workplace impairment. We report extensive data on individuals with fewer and/or milder ADHD symptoms (ASRS strata II and III comprising of >30% of the participants), a group that has not previously been studied in detail. Importantly, we report significant impairment in health, personal and social domains in this group, which often goes unnoticed. In addition, we report results for continuous measures of ADHD symptoms, which allows us to distinguish between inattention and hyperactivity related impairment. Finally to better understand the co-occurrence between ADHD and depression/anxiety symptoms we clearly show how the regression results were affected by the inclusion or exclusion of depression/anxiety symptoms in the model. This comparison aids in gauging the unique contribution of ADHD symptoms to impairment.

This study has several limitations. First, information on ADHD symptoms during childhood was unavailable and hence we could not assess participants clinically using the current diagnostic criteria. Similar limitations also apply to measures of depression and anxiety symptoms, which were not diagnosed in clinical terms. Nevertheless, the associations we observed between ADHD symptoms and functional impairment was very similar to those reported previously in both patient and population samples, albeit in a younger age group. Second, all measures were based on self-reports, which might not be completely accurate (e.g. social desirability and current emotional state could introduce biases [50], [51]). However, the assessment instruments we used in this study were reported to have good sensitivity and specificity, have been validated in different cultural settings and have been used in several reported studies [37]–[43].

In conclusion, we observed that a significant proportion of middle-aged individuals in the general population report impairment associated with ADHD symptoms – especially inattention symptoms with both males and females being equally affected. Irrespective of whether or not these individuals meet all the criteria required for a clinical diagnosis of ADHD, the significant functional impairment and depression/anxiety symptoms they experience clearly demonstrates that the presence of these symptoms constitutes a substantial burden for middle-aged adults. For societies with a large aging population this burden, which is currently unrecognized, is likely to result in significant costs for individuals as well as the community. Impairment associated with ADHD symptoms is responsive to interventions in children, adolescents and young adults [52]. There is, therefore, a good prospect that suitable intervention would reduce the substantial negative impact of ADHD symptoms on health and quality of life in older age groups that we have demonstrated here. Thus, there is a compelling need to develop a greater understanding of the presentation and impact of ADHD in later life and to develop age-appropriate approaches to diagnosis and treatment.
